# Evaluation of the Swabbing of Disposable Absorbent Incontinence Products for Assessing the Carriage of Multiresistant Enterobacteriaceae in Nursing Home Residents

**DOI:** 10.3389/fmicb.2017.01858

**Published:** 2017-09-29

**Authors:** Alexis Naf, Marie Decalonne, Sandra Dos Santos, Laurent Mereghetti, Nathalie L. van der Mee-Marquet

**Affiliations:** ^1^Cellule Régionale d’Epidémiologie Nosocomiale, Hôpital Trousseau, Service de Bactériologie, Virologie, Hygiène, Centre Hospitalier Régional Universitaire, Tours, France; ^2^Centre d'Appui pour la Prévention des Infections Associées aux Soins pour la région Centre Val de Loire, Hôpital Bretonneau, Service de Bactériologie, Virologie, Hygiène, Centre Hospitalier Régional Universitaire, Tours, France; ^3^Unité de bactériologie, Hôpital Bretonneau, Service de Bactériologie, Virologie, Hygiène, Centre Hospitalier Régional Universitaire, Tours, France

**Keywords:** nursing homes, multi-resistant bacteria, asymptomatic carriage, ESBL-producing enterobacteriaceae, carbapenemase-producing enterobacteriaceae, rectal swabbing, infection control

## Abstract

We compared the performance of incontinence product (IP) and rectal swabbing for the detection of multidrug-resistant Enterobacteriaceae (MDRE) carriage in a large multicenter study conducted in February 2017 among the residents of 23 French nursing homes. The study included 547 residents who habitually wore IP, 88 of whom were MDRE carriers (16.1%). Positive results were obtained for both rectal and IP swabs for 64 of these residents, for rectal swabs only for 22 and for IP swabs only for two of these patients. The estimated prevalence of MDRE carriage depended on the type of sample: 15.7% for rectal swabs and 12.1% for IP swabs (*p* < 0.001). The positive percent agreement was 84.2% and the negative percent agreement was 97.4%. Rectal swabbing remains the best method for detecting MDRE carriage in elderly residents, but our findings provide support for the use of swabs from IP used overnight to increase response rates in MDRE surveys in elderly residents that habitually wear IP, when rectal swabbing is not feasible.

## Introduction

The increasing incidence of bloodstream infections caused by extended-spectrum B-lactamase-producing Enterobacteriaceae (ESBLE) and carbapenemase-producing Enterobacteriaceae (CPE) is a source of concern and multidrug-resistant Enterobacteriaceae (MDRE) have become a major public health problem.

MDRE carriage among nursing home (NH) residents is favored by the frequent use of broad-spectrum antibiotics and the lax application of standard precautions during care activities in these institutions (Montoya et al., 2016). Colonized residents may serve as vectors, transferring MDRE to hospitals, where these bacteria may spread to highly vulnerable patients (Lee et al., 2013). Prevention of the uncontrolled spread of MDRE in NHs is thus of the utmost importance, and strategies likely to decrease the frequency of MDRE colonization in NHs should be encouraged.

French guidelines do not recommend the routine screening of NH residents for MDRE carriage, but they do promote the implementation of standard precautions during care, and suggest that barrier precautions should be used during the management of CPE carriers (but not ESBLE carriers). Since 2010, our regional infection control team has been running a specific program for NHs involving (i) the education of healthcare workers about the importance of rational antibiotic use, hygiene procedures, and environment cleansing, (ii) observational studies, and (iii) a once-yearly monitoring of MDRE carriage in a representative panel of residents. The overall goal of this program is to decrease antibiotic selection pressure and limit the transmission of MDRE between residents through improvements in antibiotic use and compliance with standard precautions, particularly during the care of the most dependent residents.

Observational studies and MDRE carriage surveys have shown that high MDRE carriage rates are associated with the spread of epidemics within the NH and the inadequate implementation of standard precautions (Cochard et al., 2014). MDRE carriage surveys are used to provide an indicator of local care quality and information for the adaptation of preventive strategies in NHs nationwide. Rectal swabbing is a valuable technique for investigating gut MDRE carriage in patients (Lerner et al., 2013), currently used for such studies in the NHs of our region. However, the insertion of a cotton swab into the rectum is often inacceptable to residents, who may experience discomfort during collection of the sample by a clinician, and frequently embarrassing for healthcare workers. Refusals are frequent as a result. We therefore sought an alternative solution for the surveillance of fecal MDRE carriage in residents, with the aim of improving the willingness of healthcare workers to take part in studies of MDRE carriage. We hypothesized that the swabbing of used IPs could be used to detect the MDRE colonizing the guts of incontinent residents, given (i) the frequent co-colonization of the gut and urine by MDRE in elderly individuals (Jans et al., 2013), (ii) the high prevalence of urinary and fecal incontinence among residents (Leung and Schnelle, 2008), (iii) the predominant use of single-use IPs by NH residents, (iv) the favorable (warm and moist) conditions for the growth of bacteria originating from urine and the intestinal tract provided by IPs, and (v) the likelihood of the swabbing of IPs being more acceptable to residents and healthcare workers than rectal swabbing. Accordingly, during the survey conducted in February 2017, we compared the performance of IP and rectal swabs obtained from residents in the habit of using IPs, for the detection of colonizing ESBLE and CPE in the NH setting.

## Materials and methods

### Confidentiality and ethical aspects

This study was performed in accordance with French national recommendations for the prevention of infection in healthcare. Ethics approval was obtained at national level from the *Réseau Alerte Investigation Surveillance des Infections Nosocomiales*. The study was managed jointly by the NH directors, the hygiene nurses and physicians responsible for resident care, and the regional infection control team. All nominative data were rendered anonymous in this study.

### Study population

Twenty-three NHs agreed to participate in the study (with 83–449 beds per NH, and 4,177 beds in total). All residents living in the participating NHs in February 2017 were eligible for inclusion in this study. All the residents from a single unit of each NH were included (25 or 31, depending on the NH). All residents or their relatives were individually approached and asked to consent to access to the resident's medical records and for the culture of a single fecal sample to test for ESBLE and CPE.

### Study design

The 1 day point-prevalence study was performed over a 1 month period. Each resident was included once. We used rectal swabs to screen residents for ESBLE and CPE carriage. In addition, for residents habitually wearing disposable IPs, we swabbed IPs that had been used overnight when they were removed during morning nursing care. A swab culture transport system (Transystem, Copan) was used for the rectal and IP samples. Swabs were transferred to the regional laboratory on the day of collection and processed immediately. Swabs were immersed in 1 mL sterile water to generate a suspension, 0.3 mL of which was streaked onto a Chromagar ESBL selective agar plate and a mSuperCarba selective agar plate (Chromagar, France). The plates were incubated for 48 h at 35°C. Colony-forming units (CFUs) were identified by MALDI-TOF (Bruker, France) and tested for ESBL production in a double-disk synergy test, in accordance with French national recommendations. Bacteria displaying intermediate susceptibility or resistance to ertapenem (MIC > 0.5 mg/L) and/or imipenem (MIC >2 mg/L) were tested for CPE with the Carba NP test (Nordmann et al., 2012).

### Data collection

For all the NH residents studied, a standardized questionnaire was used to collect demographic data (age and sex), and information about physical disability, urinary and fecal incontinence, and significant comorbid conditions. Various risk factors for the carriage of MDR bacteria were also recorded: hospitalization and antibiotic use (clavulanate-amoxicillin, third-generation cephalosporins, fluoroquinolones, carbapenems, vancomycin) within the 6 months before inclusion in the study.

### Statistical analysis

All variables included in the univariate analysis, with McNemar's test, as appropriate. All statistical tests were two-tailed. Values of *P* < 0.05 were considered to be statistically significant.

## Results

We selected 701 residents from 23 different NHs for inclusion: 21 declined participation (3.0%), so 680 residents in total were enrolled in the study and screened for MDRE carriage.

### Population characteristics

The population of 680 residents enrolled in this study consisted of 515 women (75.7%) and 165 men (24.3%) between the ages of 51 and 108 years (mean age, 87 years) (Table 1). Many were in a poor state of health (71.2% were unable to walk unassisted), and 61.0% of the residents had a McCabe index of 1 or 2 (illness likely to prove fatal within the next 5 years). Diabetes mellitus was reported in 16.9% of the patients and 5.6% had cancer. Incontinence was frequent: urinary incontinence in 80.6% of residents and fecal incontinence in 67.9%. Recent hospitalization was noted for 20% of the enrolled residents and recent antibiotic treatment for 34.4%: 9.1% were treated with third-generation cephalosporins and 5.3% were treated with clavulanate-amoxicillin. Fluoroquinolone, imipenem, and vancomycin treatments were reported for 4.3, 0.3, and <0.1% of the residents, respectively. On the day of the study, 2.9% of the residents presented signs of infection.

**Table 1:** Demographic and clinical characteristics of residents testing positive (*n* = 99) and negative (*n* = 581) for ESBLE carriage in the 23 NHs.

**Characteristics of residents**	**Positive, *n* (%)**	**Negative, *n* (%)**	***p***
Age > 87 years	44 (44.4)	308 (53.0)	
Physical disability	73 (73.7)	411 (70.7)	
Comorbid conditions			
Diabetes mellitus	15 (15.1)	100 (17.2)	
Cancer	2 (2.0)	36 (6.2)	
McCabe 1 or 2	58 (58.6)	357 (61.4)	
Incontinence			
Urinary	87 (87.9)	461 (79.3)	0.047
Fecal	73 (73.7)	389 (66.9)	
Hospitalization in the last 6 months	24 (24.2)	110 (18.9)	
Antibiotic use in the last 6 months	46 (46.5)	188 (32.3)	0.006
Clavulanate-amoxicillin	6 (6.1)	30 (5.2)	
Fluoroquinolones	6 (6.1)	23 (3.9)	
3rd-generation cephalosporins	16 (16.2)	46 (7.9)	0.008
Vancomycin	0	1 (<0.1)	
Carbapenems	2 (2.0)	0	0.015
Infection on the day of the study	2 (2.0)	18 (3.1)	

### Carriage of ESBLE and CPE

We processed 680 rectal swabs and 547 IP swabs from the 680 residents enrolled. Visible fecal staining was observed on all rectal swabs. By contrast, most of the IP swabs were unstained. None of the residents enrolled in this study were CPE carriers. By contrast, 99 were ESBLE carriers (14.5%) (Table 1). The MDRE carriage rate ranged from 0 to 51.7%, depending on the NH. Of the 99 ESBLE isolated, 65 were identified as *Escherichia coli* (65.6%), 27 as *Klebsiella pneumoniae* (27.3%), three as *Enterobacter cloacae*, one as *Proteus vulgaris*, and three as *Citrobacter*. The antibiotic susceptibility patterns of the ESBLE are presented in Table 2. The ESBLE carriers were in a poorer state of health than non-carriers (Table 1). ESBLE colonization was associated with urinary incontinence (*p* = 0.047), recent antibiotic treatment (*p* = 0.006), and treatment with third-generation cephalosporins and carbapenem (*p* = 0.008 and 0.015, respectively).

**Table 2:** Antibiotic susceptibility pattern of the 99 ESBLSE isolates.

	Antibiotype^*^																	**Number of isolates**
*E. coli*	AMOX	TICAR	PIP	CN	CTX	ATM	CAZ	FEP			TM	NET	GM	NA	OFX	CIP	SXT	2
	AMOX	TICAR	PIP	CN	CTX	ATM	CAZ	FEP		AN	TM	NET		NA	OFX	CIP	SXT	1
	AMOX	TICAR	PIP	CN	CTX	ATM	CAZ	FEP			TM			NA	OFX	CIP	SXT	1
	AMOX	TICAR	PIP	CN	CTX	ATM	CAZ	FEP						NA	OFX	CIP	SXT	1
	AMOX	TICAR	PIP	CN	CTX	ATM	CAZ	FEP	ERM					NA	OFX	CIP	SXT	2
	AMOX	TICAR	PIP	CN	CTX	ATM		FEP			TM			NA	OFX	CIP	SXT	1
	AMOX	TICAR	PIP	CN	CTX	ATM					TM			NA	OFX	CIP	SXT	1
	AMOX	TICAR	PIP	CN	CTX						TM			NA	OFX	CIP	SXT	2
	AMOX	TICAR	PIP	CN	CTX									NA	OFX	CIP	SXT	2
	AMOX	TICAR	PIP	CN	CTX		CAZ							NA	OFX	CIP	SXT	1
	AMOX	TICAR	PIP	CN	CTX	ATM	CAZ	FEP		AN	TM	NET	GM	NA	OFX	CIP		1
	AMOX	TICAR	PIP	CN	CTX	ATM	CAZ	FEP	TM			NET	GM	NA	OFX	CIP		2
	AMOX	TICAR	PIP	CN	CTX	ATM	CAZ	FEP	TM				GM	NA	OFX	CIP		1
	AMOX	TICAR	PIP	CN	CTX			FEP	TM				GM	NA	OFX	CIP		1
	AMOX	TICAR	PIP	CN	CTX	ATM	CAZ		TM					NA	OFX	CIP		1
	AMOX	TICAR	PIP	CN	CTX	ATM	CAZ	FEP						NA	OFX	CIP		1
	AMOX	TICAR	PIP	CN	CTX			FEP						NA	OFX	CIP		2
	AMOX	TICAR	PIP	CN	CTX	ATM	CAZ	FEP						NA	OFX	CIP		10
	AMOX	TICAR	PIP	CN	CTX	ATM	CAZ	FEP	ERM					NA	OFX	CIP		1
	AMOX	TICAR	PIP	CN	CTX									NA	OFX	CIP		4
	AMOX	TICAR	PIP	CN	CTX	ATM	CAZ							NA				1
	AMOX	TICAR	PIP	CN	CTX									NA			SXT	2
	AMOX	TICAR	PIP	CN	CTX			FEP						NA			SXT	1
	AMOX	TICAR	PIP	CN	CTX		CAZ							NA				1
	AMOX	TICAR	PIP	CN	CTX									NA				1
	AMOX	TICAR	PIP	CN	CTX												SXT	2
	AMOX	TICAR	PIP	CN	CTX	ATM	CAZ										SXT	1
	AMOX	TICAR	PIP	CN	CTX	ATM	CAZ	FEP									SXT	1
	AMOX	TICAR	PIP	CN	CTX	ATM	CAZ	FEP										6
	AMOX	TICAR	PIP	CN	CTX	ATM	CAZ											1
	AMOX	TICAR	PIP	CN	CTX	ATM												1
	AMOX	TICAR	PIP	CN	CTX													5
	AMOX	TICAR	PIP	CN	CTX			FEP			TM	NET						1
	AMOX	TICAR	PIP		CTX			FEP									SXT	1
	AMOX	TICAR	PIP		CTX		CAZ											1
	AMOX	TICAR	PIP		CTX													1
*P. vulgaris*	AMOX	TICAR	PIP	CN	CTX													1
*K. pneumoniae*	AMOX	TICAR	PIP	CN	CTX	ATM	CAZ	FEP	ERM	AN	TM	NET	GM	NA	OFX	CIP	SXT	1
	AMOX	TICAR	PIP	CN	CTX	ATM	CAZ	FEP		TM	NET	GM		NA	OFX	CIP	SXT	4
	AMOX	TICAR	PIP	CN	CTX	ATM	CAZ			TM		GM		NA	OFX	CIP	SXT	1
	AMOX	TICAR	PIP	CN	CTX	ATM		FEP	ERM					NA	OFX		SXT	1
	AMOX	TICAR	PIP	CN	CTX	ATM	CAZ	FEP		TM				NA	OFX	CIP	SXT	1
	AMOX	TICAR	PIP	CN	CTX	ATM	CAZ	FEP		TM					OFX	CIP	SXT	5
	AMOX	TICAR	PIP	CN	CTX	ATM	CAZ			TM					OFX	CIP	SXT	1
	AMOX	TICAR	PIP	CN	CTX	ATM	CAZ	FEP		TM						CIP	SXT	3
	AMOX	TICAR	PIP	CN	CTX		CAZ	FEP		TM						CIP	SXT	2
	AMOX	TICAR	PIP	CN	CTX			FEP									SXT	2
	AMOX	TICAR	PIP	CN	CTX	ATM		FEP									SXT	1
	AMOX	TICAR	PIP	CN	CTX	ATM	CAZ			TM	NET			NA	OFX	CIP		1
	AMOX	TICAR	PIP	CN	CTX	ATM	CAZ	FEP						NA	OFX	CIP		4
*C. freundii*	AMOX	TICAR	PIP	CN	CTX													1
	AMOX	TICAR		CN	CTX	ATM	CAZ							NA	OFX	CIP		1
*C. koseri*	AMOX	TICAR	PIP	CN	CTX													1
*E. cloacae*	AMOX	TICAR	PIP	CN	CTX	ATM	CAZ	FEP	ERM	AN	TM	NET	GM	NA	OFX	CIP	SXT	1
	AMOX	TICAR	PIP	CN	CTX	ATM	CAZ	FEP			TM	GM						1
	AMOX	TICAR	PIP	CN	CTX	ATM	CAZ	FEP			TM	NET	GM	NA	OFX	CIP	SXT	1

* *AMOX, amoxicillin; TICAR, ticarcillin; PIP, piperacillin; CN, CTX cefotaxime; ATM, aztreonam; CAZ, ceftazidime; FEP, cefpirome; ERM, ertapenem; AN, amikacin; TM, tobramycin; NET, netilmicin; GM, gentamicin; NA, nalidixic acid; OFX, ofloxacin; CIP, ciprofloxacin; SXT, trimethoprim-sulfamethoxazole*.

### Comparison of incontinence product and rectal swabbing for the detection of ESBLE and CPE carriage

**Figure 1:**
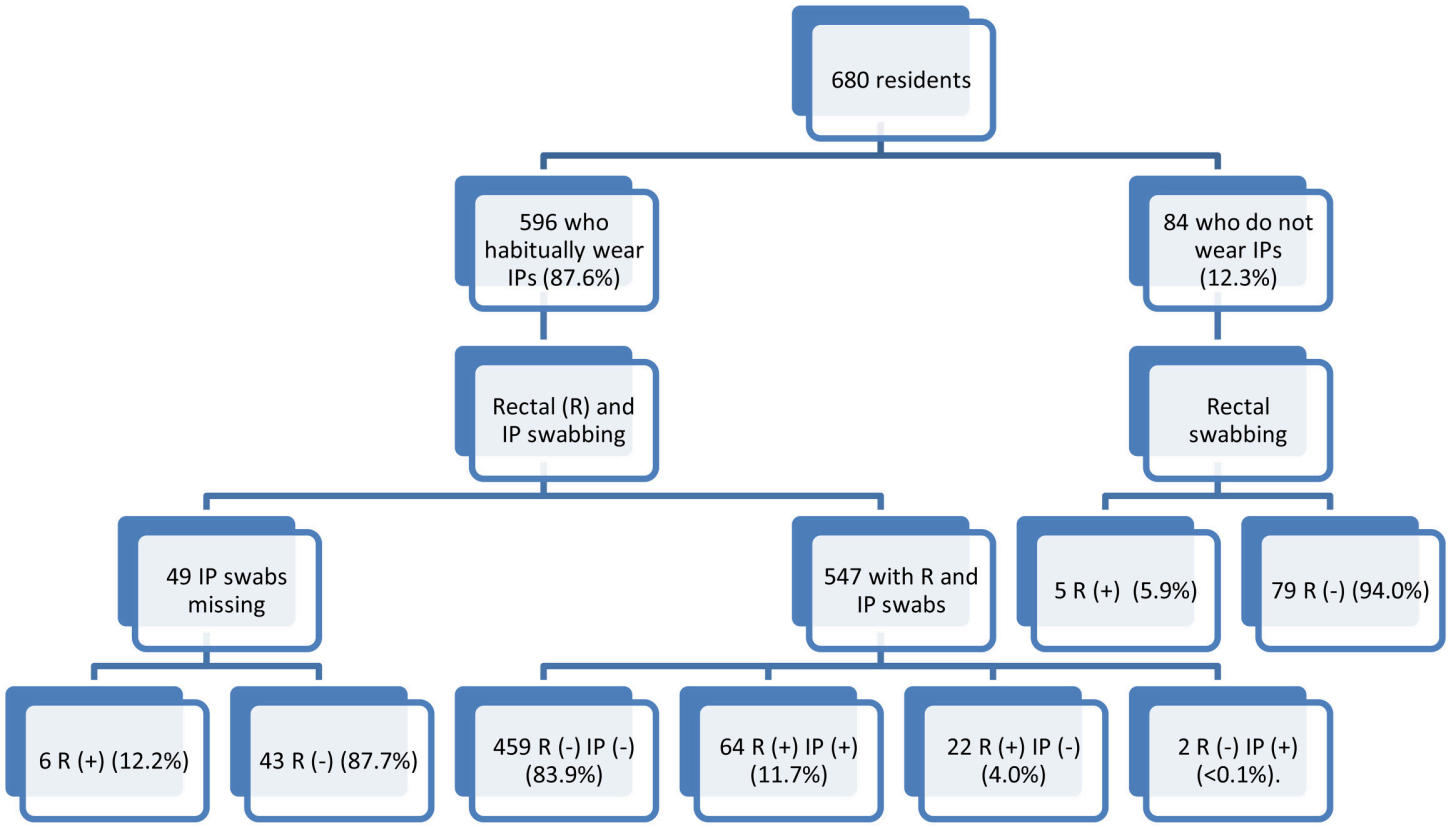
Distribution of positive and negative rectal and IP swabs.

In total, 596 of the 680 residents enrolled habitually wore IPs (87.6%), whereas the remaining 84 did not (Figure 1). The 84 residents who did not wear IP underwent rectal swabbing only. Both rectal and IP swabbings were performed for 547 of the 596 residents who used IPs. For the other 49 residents routinely using IPs, only a rectal swab was obtained, due to the healthcare worker forgetting to swab the IP. We compared the performance of rectal and IP swabbing, using the data obtained for the 547 residents for whom both types of swab were obtained (Figure 1): 459 residents (83.9%) were non-carriers, with negative results obtained for both swabs, and 88 were considered to be carriers (16.1%), based on the obtainment of a positive result for a rectal and/or IP swab (Table 3). The estimated prevalence of ESBLE carriage depended on the type of sample used. In 72.7% of cases, positive carriage was detected with both swabs. By contrast, 22 carriers were identified on the basis of positive rectal swabs but their IP swabs were negative, and two carriers were identified on the basis of positive IP swabs but had negative rectal swabs. There was a statistically significant difference in performance between the two sampling methods (McNemar Chi Squared 16.7; *p* < 0.001), in favor of rectal swabbing. The positive percent agreement was 84.2% and the negative percent agreement was 97.4%.

**Table 3:** Comparison of the ESBLE carriage detection rates obtained with rectal (R) and incontinence product (IP) swabs.

**NH**	**Number of residents wearing IPs (*****n*** = **590)**					**% ESBLE carriage rate with only**	
	***n***.	**R (−)**	**R (+)**	**R (+)**	**R (−)**	**R**	**IP**
		**IP (−)**	**IP (+)**	**IP (−)**	**IP (+)**		
nh3280	25	25				0	0
nh143	29	28		1		3.4	0
nh3378	25	24		1		4.0	0
nh3247	23	22	1			4.3	4.3
nh3245	21	20	1			4.8	4.8
nh3272	19	18	1			5.3	5.3
nh3335	27	25	1	1		7.4	3.7
nh3175	26	24	1	1		7.7	3.8
nh136	23	21	2			8.7	8.7
nh3316	18	16	2			9.7	0
nh3297	20	18	2			10.0	10.0
nh3066	17	15	2			11.8	11.8
nh3127	27	23	2	2		14.8	7.4
nh3073	26	22	3	1		15.4	11.5
nh3315	25	20	3	1	1	16.0	16.0
nh3369	25	21	3	1		16.0	12.0
nh3340	30	25	4	1		16.7	13.3
nh3167	25	20	4	1		20.0	16.0
nh3114	22	17	4	1		22.7	18.2
nh3097	16	12	2	2		25.0	12.5
nh3327	26	19	4	3		26.9	15.4
nh3162	28	14	11	2	1	46.4	42.9
nh3111	24	10	13	1		58.3	54.2
All	596	459	64	22	2	15.7	11.1

The MDRE isolates obtained from 64 residents with both rectal and IP swabs had similar antibiotic susceptibility patterns, highly suggestive of clonality in all cases. Most of the MDRE cultures obtained from rectal or IP swabs in these cases were of major importance. By contrast, the MDRE cultures for the 22 cases of MDRE carriage identified on the basis of rectal swabs only were mostly much lower, suggesting low-level MDRE carriage.

## Discussion

We report the results from a large-scale multicenter study of ESBLE and CPE carriage among elderly French NH residents (*n* = 680). None of the residents tested was carrying CPE, but we detected ESBLE carriage in 14.5% of the residents enrolled, at rates of 0–58.3%, depending on the NH considered. These findings indicate that CPE have not yet spread to the NHs of our region, but they are consistent with an epidemic spread of ESBLE in a third of the NHs, justifying a continuation of current infection control efforts. The factors associated with high-risk MDRE carriage among residents were those identified in previous studies (i.e., urinary incontinence and recent treatment with a broad-spectrum antibiotic) (Cochard et al., 2014; Montoya et al., 2016).

We provide here the first evaluation of IP swabbing as a possible alternative to rectal swabbing for the detection of MDRE in NH residents. The results for this large population of residents from 23 different NHs indicate that rectal swabbing remains the best method for the detection of MDRE carriers. Rectal swabbing should be preferred during investigations of outbreaks, for which maximal sensitivity is required. The reasons for the lower sensitivity of IP swabbing than of rectal swabbing remain to be identified. There are three possible explanations: (1) an inoculum effect, as the detection of minor MDRE carriage by rectal swabbing was associated with an absence of carriage detection by IP swabbing; (2) the absence of fecal material on some IP swabs, and (3) the presence of antimicrobial agents in the IP that might have interfered with the colonizing bacteria. However, this seems unlikely, as cases of MDRE carriage detected by rectal swabbing but not by IP swabbing were detected in the same NHs as cases of MDRE carriage identified by IP swapping, and all the residents of these NHs wore similar IPs. Further studies are required to investigate this issue further.

However, imperfect, our study indicates that IP swabbing has a satisfactory sensitivity for the detection of MDRE carriers among incontinent residents. Given the need to increase the acceptability of fecal carriage surveys to residents and healthcare workers, our findings provide support for the use of IP swabs for epidemiological surveys as an alternative method for detecting MDRE carriage in NHs, when rectal swabbing is not feasible. In such cases, IP swabbing practices should be strictly controlled. First, IP swabbing should only be performed for elderly residents who habitually wear IPs due to urinary and/or fecal incontinence. Second, the time of contact between the perineal area and the IP should be as long as possible, to make it possible to collect sufficient urine and/or feces for testing. In practice, swabbing should be restricted to the IP used overnight and performed when the IP is replaced in the morning.

## Conclusion

MDRE surveillance is a priority for NHs, together with increasing the awareness, among healthcare workers, of the importance of MDRE as a public health issue. The place of MDRE carriage surveys within the infection control studies of NHs has yet to be defined. We believe that, in addition to observational studies and educational programs, such surveys are a promising tool for benchmarking and improving the compliance of healthcare workers in NHs with practices preventing the spread of MDRE. For incontinent residents, MDRE carriage surveys will probably be improved by the possible use of IP swabbing when rectal swabbing is not feasible, as this will probably increase participation.

## Author contributions

AN and SS did the technical work. MD and NvdM analyzed data. NvdM designed the study and wrote the MS. LM helped for redaction of the MS.

## List of the members of the infection control group

P. Amirault (Vierzon), R. Andriamiarisoa (Neuville aux Bois), M. Audibert (Gien), F. Barbier (St. Amand Montrond), E. Bardon (Selles sur Cher), H. Bazzi (beaugency), N. Begat (Luynes), B. Benaicha (Pithiviers), D. Bigard (La Loupe), A. Blondeau (Chateaudun), S. Bordeaux (Montrichard), S. Cescutti (neuville aux Bois), B. Clavier (Patay), N. Clisson (Fleury les Aubrais), O. David (Fleury les Aubrais), P. De Calbiac (Romorantin, St. Aignan sur Cher), P. De person (Sully sur Loire), C. Descormiers (Luynes), P. Despres (St. Amand Montrond), M. Diard (Sully sur Loire), E. Dubost (Chatillon sur Indre), D. Dureuil (Selles sur Cher), A. Dutey (Chatillon sur Indre), C. Etienne (Tours), A. Faudon-Gibelin (Chartres), D. Gallopin (Fleury les Aubrais), D. Gauthier (Sully sur Loire), C. Godfrin (Neuville aux Bois), D. Guenot (St. Amand Montrond), M. Haberbusch (Chateaudun), S. Jouanneau (St. Aignan sur Cher), JM. Laurelli (Gien), O. Lehiani (Vierzon, St. Amand Montrond), A. Lemore (Montoire sur le Loir), P. Leroy (St. Aignan sur Cher), MC. Lidon (Romorantin), S Maintier (Montoire sur le Loir), MJ Malepart (Chateaudun), JL. Marteaux (Le Blanc), V. Michel (Le Blanc), D. Montoy (Bourges), V. morange (Tours), M. Morvan (La Châtre), L. Ologoudou (La Châtre), M. Peris (Tours), G. Petit le Gouas (Chartres), A. Philippe (Luynes), C. Pierlot (Patay), MC. Pocquet (Pithiviers), M. Prevost-Oussar (Pithiviers), R. Quentin (Tours), D. Ratovohery (Chateauroux), S. Renard (Montoire sur le Loir), B. Rousseau (Gien), A. Severin (Vierzon), A. Stalter (Beaugency), M. Tarsac (Bourges).

### Conflict of interest statement

The authors declare that the research was conducted in the absence of any commercial or financial relationships that could be construed as a potential conflict of interest.
